# Infection or Contamination with *Rothia, Kocuria, Arthrobacter* and *Pseudoglutamicibacter—*a Retrospective Observational Study of Non-*Micrococcus Micrococcaceae* in the Clinic

**DOI:** 10.1128/jcm.01484-22

**Published:** 2023-03-22

**Authors:** Görel Odeberg, Anna Bläckberg, Torgny Sunnerhagen

**Affiliations:** a Division of Infection Medicine, Department of Clinical Sciences Lund, Lund University, Lund, Sweden; b Department of Infectious Diseases, Skåne University Hospital, Lund, Sweden; c Clinical Microbiology and Disease Control, Region Skåne Office for Medical Services, Lund, Sweden; d Department of Clinical Microbiology, Rigshospitalet Copenhagen University Hospital, Copenhagen, Denmark; Pattern Bioscience

**Keywords:** Arthrobacter, bacteremia, bacterial infections, blood culture, catheter-related infections, infectious disease medicine, Kocuria, Micrococcaceae, Pseudoglutamicibacter, Rothia

## Abstract

Rothia, Kocuria, Arthrobacter, and Pseudoglutamicibacter are bacterial species within the family *Micrococcaeae*. Knowledge of human infections due to these bacteria is limited. This study aimed to examine features of infections caused by non-Micrococcus *Micrococcaeae* (NMM). Findings of NMM from blood cultures and other sterile cultures from 2012 to 2021 were identified from the records of the Department of Clinical Microbiology in Region Skåne, Lund, Sweden. Medical records were retrospectively reviewed. True infection was defined as having signs of infection, no other more likely pathogen, and no other focal infection, together with two positive blood cultures or one positive blood culture and an intravascular device. A total of 197 patients with findings of NMM in blood cultures were included. Among adult patients with bacteremia, 29 patients (22%) were considered to have a true infection. Adults with true infection were significantly more likely to have malignancy (69%), leukopenia (62%), and treatment with chemotherapeutics (66%) compared to patients with contaminated samples (24%, 3%, and 8%, respectively) (*P < *0.001). A total of 31 patients had findings of NMM in other sterile cultures, and infections were considered true in joints (*n *= 4), a pacemaker (*n *= 1), and peritoneal dialysis fluid (*n *= 1). Infections due to NMM occur but are rare. Growth of NMM in blood cultures should be suspected to be a true infection mainly in immunocompromised patients.

## INTRODUCTION

*Actinobacteria* (or *Actinomycetota*) make up one of the largest taxonomic units within the bacteria domain. It is a diverse group of Gram-positive bacteria since it contains pathogens, soil inhabitants, and bacteria within the normal flora in humans and animals (1, 2). *Micrococcaceae* is a family within the phylum *Actinobacteria*, containing, among other genera, Micrococcus, Rothia, Kocuria, Arthrobacter, and Pseudoglutamicibacter (3).

The bacteria genus Rothia was first introduced in 1967 by Georg and Brown (4) and has its natural habitat in the oral cavity of humans. Few case reports are published of human infections due to Rothia and have encompassed infective endocarditis, pneumonia, meningitis, and bacteremia. Since Rothia is part of the normal flora in humans, findings of Rothia from blood cultures may often be considered contamination rather than an invasive infection (5–8).

In 1995, a group of bacteria within the genus Micrococcus was reclassified as a separate genus, named Kocuria (9). It inhabits the skin and oral cavity of humans and is an uncommon cause of infection. Therefore, it is often dismissed as contamination when there is growth in blood cultures. Nevertheless, case reports have described infections such as urinary tract infections, peritonitis, and infective endocarditis due to Kocuria (10–12). These infections often occurred in patients with predisposing factors such as chronic catheterization, malignancies, and end-stage renal disease with dialysis (13).

Arthrobacter is a heterogenous genus of bacteria species distributed in the environment, especially soil. In contrast to Rothia and Kocuria, it does not naturally inhabit humans (14). There are only a few case reports describing human infections due to Arthrobacter, in which patients with leukemia or injection drug use have been affected (15–17).

Pseudoglutamicibacter was formerly defined as a member of the genus Arthrobacter. However, alongside updated taxonomy, the species is now defined as an own genus (18). According to earlier publications, it has low pathogenic potential. There are no cases describing severe infections in humans caused by Pseudoglutamicibacter, but there is one case with urinary tract infection (19).

Since there is a lack of studies on the clinical implications of finding these bacteria (henceforth referred to as non-Micrococcus *Micrococcaceae* [NMM]), this study aimed to investigate the microbiological and clinical aspects of infections with Rothia, Kocuria, Arthrobacter, and Pseudoglutamicibacter and whether infections due to the bacteria are more likely to occur in certain groups of patients.

## MATERIALS AND METHODS

**FIG 1:**
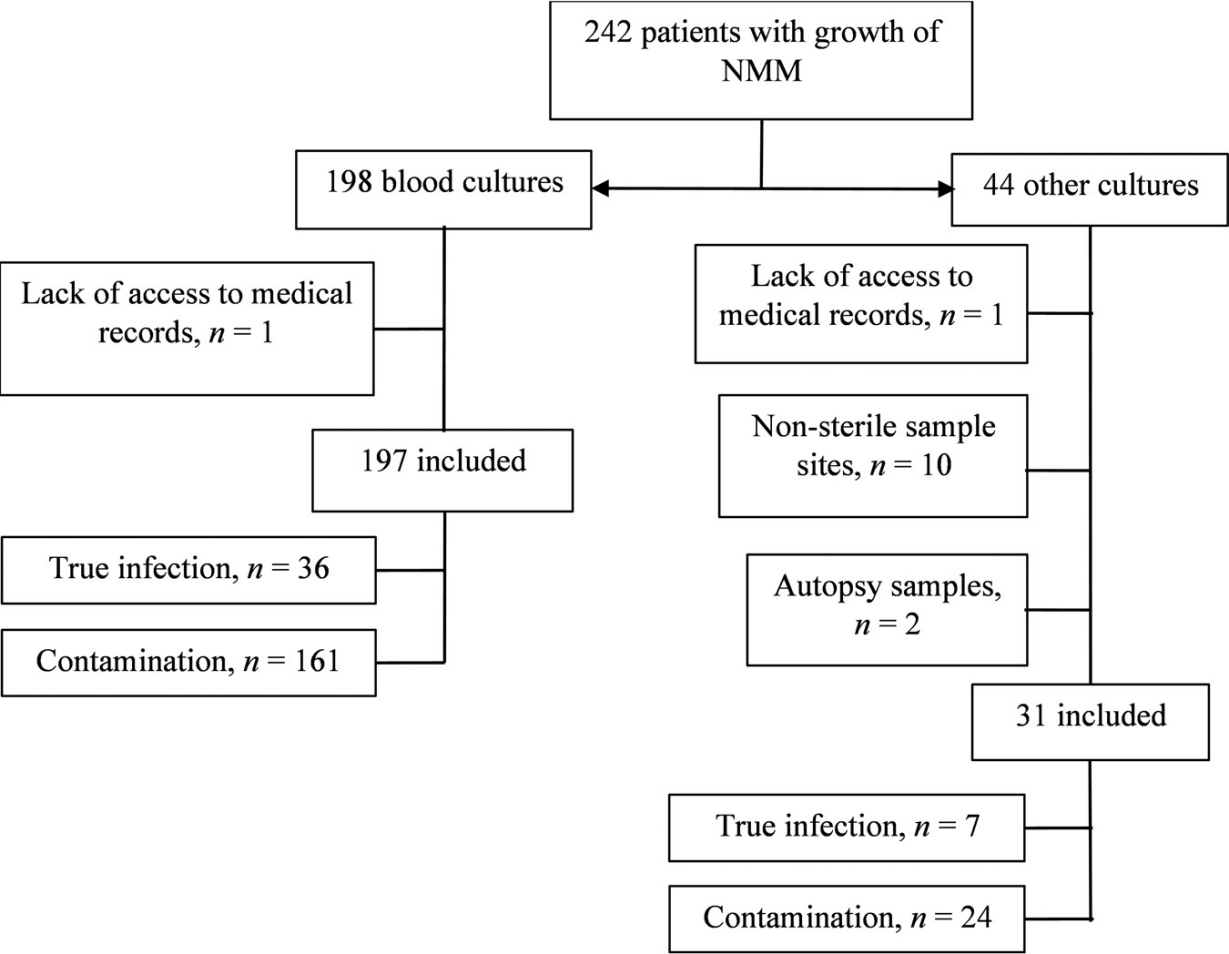
Flowchart of inclusion and exclusion.

Patients with growth of NMM in blood cultures and other sterile cultures between 2012 and 2021 were identified by searching in the laboratory information system of the clinical microbiology laboratory of Region Skåne, Lund, Sweden. This clinical microbiology laboratory is the only one in the region and serves both public and private clinics (both in- and outpatient), including primary health care and secondary and tertiary hospitals with a covered population of approximately 1.4 million. The medical records were retrospectively reviewed with the inclusion criteria being the presence of the above-mentioned bacteria in cultures. The exclusion criteria were patients who had in-hospital care outside the region and patients whose medical records were unavailable (Fig. 1). Clinical data according to a prespecified protocol involving age, bacterial species, polymicrobial growth, use of immunosuppressive treatment or chemotherapeutics, Charlson Comorbidity Index (20), fever, and leucocytosis within 48 h from when the sample was taken and presence of any intravascular device or joint prosthesis were collected.

The reason for including these specific bacteria (Rothia, Kocuria, Arthrobacter, and Pseudoglutamicibacter) is that they are the most commonly found *Micrococcaceae* in our setting, except for Micrococcus, which was excluded from the study because it is found so much more often that the inclusion was thought likely to lead to a total domination of statistical analyses.

Nosocomial infection was defined as having a positive microbiological culture after at least 48 h of hospitalization. The definition of a health care-associated infection was reported as stated by Friedman et al. (21), meaning an infection in a patient from a nursing home, who got medical care at home, who had received intravenous therapy, or who had been hospitalized within 90 days before the microbiological culture was taken. To classify sepsis and septic shock, the Sepsis-3 criteria were applied (22) and evaluated using the Sequential Organ Failure Assessment (SOFA) score (23).

### Microbiological methods.

During the study period, matrix-assisted laser desorption/ionization time-of-flight mass spectrometry (MALDI-TOF MS: Bruker Daltonics, using the Bruker MBT Compass library version most recent at the time of sample analysis) was used as the main method for bacterial species identification at the clinical microbiology laboratory, with 16S sequencing using Sanger methodology being used as the primary back-up method for some selected cases in which no species identification was possible using MALDI-TOF MS.

The blood culture system used was BacT/Alert 3D (bioMérieux) until December 2012, and Bactec FX (Becton Dickinson) after that, both using a 5-day incubation standard and using both aerobic and anaerobic bottles. For samples taken from sterile sites such as cerebrospinal fluid, joint fluid, or biopsy samples, the standard method used included direct plating samples on blood agar incubated in a CO_2_-enriched atmosphere, and fastidious aerobe agar incubated under anaerobic conditions, as well as inoculating a part of the sample in tryptic soy broth and brain heart infusion for samples originating from the central nervous system, incubated in CO_2_-enriched atmosphere. All incubations were done at 37°C, with the samples being incubated for a total of 7 days as standard. Additional plates and broth were added at the discretion of the laboratory technicians and clinical microbiologists responsible for the analysis in selected cases and for specific diagnostic needs. Sterile nonblood fluids such as joint fluid were sometimes injected directly into blood culture bottles by the physician taking the sample and were in those cases handled in the same way as blood cultures in the laboratory.

### Definition of true infection.

To determine true infection, the definition used in this study was based on criteria established for true infection with Corynebacterium and Cutibacterium (24, 25). In cases with two or more positive blood culture sets the following criteria had to be fulfilled: (i) infection confirmed by fever, hypotension, chills, or leucocytosis; (ii) no other more likely pathogenic bacteria in the bloodstream; and (iii) no other focal infection explaining the symptoms. Other focal infection was defined by two of the following: (i) isolation of a bacteria in the focal site, (ii) infection suggested by imaging results, and (iii) typical symptoms of a focal infection.

In cases with only one positive blood culture, the criteria above had to be fulfilled, and in addition, an intravascular device 48 h prior to the sample or bacteria cultured from the site of infection must be present. Due to the use of pediatric blood culture bottles with only one bottle used, for patients under 18 years of age, the definition was modified so that only one positive blood culture was needed if the treating physician interpreted it as an infection with the bacteria studied. These criteria are summarized in Table 1. Blood cultures not matching these criteria were classified as probable contamination.

**TABLE 1:** Definition of true infection

Criteria	Definition
In cases with two positive blood cultures	
Criterion 1	Infection confirmed by temp (>38°C) OR hypotension (systolic blood pressure <100 mm Hg) OR chills OR leukocytosis (>12 × 10^9^/L)
Criterion 2	No other more likely pathogen in blood cultures
Criterion 3	No other focal infection
In cases with one positive blood culture	
Criterion 4	Foreign intravascular device^*a*^ present >48 h prior to blood sample AND all of the above
In cases with patients of <18 yrs	
Criterion 5	Infection interpreted as caused by *Actinobacteria* by treating physician AND all of criteria 1 to 3

a Intravascular devices include implantable cardioverter defibrillators (ICDs), pacemakers, vascular grafts, central venous catheters, and dialysis catheters.

For other sterile cultures, criteria 1 to 3 in Table 1 were used, with the same modification as was made for patients under 18 years of age. If another more likely explanation for the symptoms was found, it was not classified as a true infection.

### Statistical analysis.

Statistical analyses were made in SPSS Statistics version 28. For categorical data, the 1chi-square test was used. For continuous variables, the Mann-Whitney *U* test was used. A *P* value <0.05 was regarded as statistically significant.

### Ethical considerations.

Due to the retrospective, observational, nature of the study, the need for patient consent was waived by the Swedish Ethics Authority (approval 2021-03354).

## RESULTS

### Description of the cohort.

A total of 197 cases of findings of NMM from blood cultures and 31 from other sterile cultures were included. Fig. 1 is a flowchart summarizing the inclusion and exclusions of patients.

### Clinical features of infections with growth of NMM in blood cultures in adults.

Characteristics of adult patients and their infections are listed in Table 2. Using the criteria in Table 1, 26 of these patients (19%) were classified as having a true infection. Adult patients having a true infection were found to be younger but also had a higher portion of malignancy (69%), leukopenia (62%), use of chemotherapeutics (66%), and nosocomial infections (52%) compared to patients who were considered to have a contamination (24%, 3%, 8%, and 15%, respectively; *P < *0.001).

**TABLE 2:** Clinical and microbiological features of adult patients with non-Micrococcus *Micrococcaeae* bacteremia^*a*^

Characteristics	Total (*n* = 152)	True infection (*n* = 29)	Contamination (*n* = 123)	*P* value of difference^*b*^
Age (yr)	71 (63 to 83)	65 (57 to 75)	72 (66 to 85)	0.005
Women	74 (49)	11 (38)	63 (51)	0.140
Charlson score	2 (1 to 4)	2 (2 to 4)	2 (1 to 4)	0.323
Malignancy	49 (32)	20 (69)	29 (24)	<0.001
Site of acquisition				
Community	70 (46)	3 (10)	67 (55)	<0.001
Nosocomial	33 (22)	15 (52)	18 (15)	<0.001
Health care-associated	49 (32)	11 (38)	38 (31)	0.466
Use of chemotherapy	29 (19)	19 (66)	10 (8)	<0.001
Immunosuppressive therapy	32 (21)	8 (28)	24 (20)	0.337
Sepsis or septic shock^*c*^	16 (10)	2 (7)	14 (11)	0.479
Intravascular device^*d*^	51 (34)	23 (79)	28 (23)	<0.001
Days of hospitalization as from blood sample taken	7 (3 to 14)	13 (7 to 22)	7 (3 to 12)	0.003
Fever^*c*^	102 (67)	25 (86)	77 (63)	0.015
Leukocytosis^*c*^	64 (42)	2 (7)	62 (50)	<0.001
Leukopenia^*c*^	22 (15)	18 (62)	4 (3)	<0.001
Mortality				
In hospital	15 (10)	3 (10)	12 (10)	0.924
Within 90 days	18 (12)	4 (14)	14 (11)	0.718
Multiple positive blood cultures	22 (14)	15 (52)	7 (6)	<0.001
Polymicrobial growth	68 (45)	2 (7)	66 (54)	<0.001
Other focal infection	66 (43)	0 (0)	66 (54)	<0.001
Time to positivity (h)^*e*^	35 (25 to 57)	26 (16 to 36)	44 (31 to 64)	0.002
Genus and species				0.383 (genus), 0.220 (species)
Rothia	108 (71)	24 (83)	84 (68)	
R. dentocariosa	26 (17)	2 (7)	24 (20)	
R. mucilaginosa	53 (35)	16 (55)	37 (30)	
R. aeriae	1 (1)	0 (0)	1 (1)	
Rothia spp. not further specified	28 (18)	6 (21)	22 (18)	
Kocuria	28 (18)	4 (14)	24 (20)	
K. kristinae	2 (1)	0 (0)	2 (2)	
Kocuria spp. not further specified	26 (17)	4 (14)	22 (18)	
Arthrobacter	10 (7)	1 (3)	9 (7)	
Pseudoglutamicibacter	6 (4)	0 (0)	6 (5)	

a Categorial data are presented as No. (%). Continuous variables are presented as medians (interquartile ranges). For categorial data, the chi-square test is used. For continuous data, the Mann-Whitney *U* test is used. Adults are defined as patients ≥18 years of age.

b Difference between those with true infection and those with contaminated samples.

c Within 48 h from when the blood sample was taken.

d Intravascular devices include implantable cardioverter defibrillators (ICDs), pacemakers, vascular grafts, central venous catheters, and dialysis catheters.

e Only for those with monomicrobial growth (*n* = 39).

Looking at the number of positive blood cultures, 22 patients (15%) had more than one positive blood culture. Of these, 14 were infected with some species of Rothia. The 22 patients with multiple positive blood cultures were found to have a higher incidence of malignancy (68%), leukopenia (50%), use of chemotherapeutics (55%), and nosocomial infections (45%) compared those atients with growth of NMM in only one culture (26%, 8%, and 13% [*P < *0.001] and 18% [*P = *0.003], respectively).

### Clinical features of infections with growth in blood cultures of NMM in children.

Characteristics of patients < 18 years of age and their infections are listed in Table 3. Among these, 7 patients (16%) were classified as having a true infection, and 38 (84%) had a contaminated blood sample. Children with a true infection were found to have higher Charlson score (*P = *0.010), a higher incidence of malignancy (71%), use of chemotherapeutics (71%), leukopenia (71%), and nosocomial infections (71%) compared to those children with a contaminated blood cultures (8%, 8%, 5%, and 29%, respectively; *P < *0.001).

**TABLE 3:** Clinical and microbiological features of children with non-Micrococcus *Micrococcaeae* bacteremia^*a*^

Characteristics	Total (*n* = 45)	True infection (*n* = 7)	Contamination (*n* = 38)	*P* value of difference^*b*^
Age (yr)	1 (0 to 5)	3 (0 to 8)	1 (0 to 3)	0.362
Women	22 (49)	3 (43)	19 (50)	0.728
Charlson score	0 (0 to 2)	2 (0 to 2)	0 (0 to 0)	0.010
Malignancy	8 (18)	5 (71)	3 (8)	<0.001
Site of acquisition				
Community	14 (31)	0 (0)	14 (37)	0.053
Nosocomial	16 (36)	5 (71)	11 (29)	0.031
Health care-associated	15 (33)	2 (29)	13 (34)	0.771
Use of chemotherapy	8 (18)	5 (71)	3 (8)	<0.001
Immunosuppressive therapy	8 (18)	2 (29)	6 (16)	0.416
Sepsis or septic shock^*c*^	0 (0)	0 (0)	0 (0)	
Intravascular device^*d*^	6 (13)	2 (29)	4 (11)	0.197
Days of hospitalization as from blood sample taken	5 (2 to 8)	6 (4 to 15)	5 (2 to 8)	0.126
Fever^*c*^	28 (62)	6 (86)	22 (58)	0.163
Leukocytosis^*c*^	14 (31)	0 (0)	14 (37)	0.053
Leukopenia^*c*^	7 (16)	5 (71)	2 (5)	<0.001
Mortality				
In hospital	2 (4)	1 (14)	1 (3)	0.169
Within 90 days	3 (7)	2 (29)	1 (3)	0.011
Multiple positive blood cultures	0 (0)	0 (0)	0 (0)	
Polymicrobial growth	12 (27)	2 (29)	10 (26)	0.901
Other focal infection	21 (47)	0 (0)	21 (55)	0.007
Time to positivity (h)^*e*^	31 (20 to 49)	25 (14 to 38)	35 (21 to 51)	0.126
Genus and species				0.466 (genus), 0.050 (species)
Rothia	38 (84)	7 (100)	31 (82)	
R. dentocariosa	9 (20)	0 (0)	9 (24)	
R. mucilaginosa	21 (47)	7 (100)	14 (37)	
Rothia spp. not further specified	8 (18)	0 (0)	8 (21)	
Kocuria	6 (13)	0 (0)	6 (16)	
Arthrobacter	1 (2)	0 (0)	1 (3)	

a The categorial data are presented as No. (%). Continuous variables are presented as medians (interquartile ranges). For categorial data, the chi-square test was used. For continuous data, the Mann-Whitney *U* test was used. Children are defined as patients <18 years of age.

b Difference between those with true infection and those with contaminated samples.

c Within 48 h from when the blood sample was taken.

d Intravascular devices include implantable cardioverter defibrillators (ICDs), pacemakers, vascular grafts, central venous catheters, and dialysis catheters.

e Only for those with monomicrobial growth (*n* = 19).

### Microbiological features of infections with growth of NMM in blood cultures.

Rothia was the most common genus, with R. mucilaginosa being the most common species, both among true infections (*n *= 23, 64%) and in total (*n *= 74, 38%). R. mucilaginosa was also the most pathogenic species, with 31% of the positive blood cultures being defined as true infections.

Median time to positivity (TTP) was statistically significantly shorter in patients who were considered to have a true infection compared with those who had contamination, 26 h (interquartile range [IQR], 16 to 36) versus 44 h (IQR, 31 to 64) (*P < *0.001) (Table 2). Identification was performed using MALDI-TOF in all cases except for four, where 16S sequencing using Sanger methodology was used.

### Severe infection with bacterial growth of NMM in blood cultures.

One patient was diagnosed with infective endocarditis caused by R. mucilaginosa, which was isolated in three sets of blood cultures, as well as on tissue from his prosthetic cardiac valve. Transthoracic echocardiogram showed a vegetation on the aortic prosthetic valve measuring 13 mm, with a suspicion of a root abscess. The patient was initially treated conservatively but then, after a cardiac arrest, underwent acute surgery, with a successful outcome after continued treatment with benzylpenicillin and gentamicin, with vancomycin being used before blood culture results were finished.

### Infections with NMM identified by cultures other than blood cultures.

A total of 31 cultures were taken from locations other than the bloodstream, with joint fluid being the most common. In total, 7 cases of true infection were found among these patients. Characteristics of patients with growth of NMM in joint fluid and bone tissue are listed in Table 4, and the characteristics of patients with growth of NMM in samples other than blood, joint fluid, and bone are listed in Table 5.

**TABLE 4:** Clinical and microbiological features of patients with non-Micrococcus *Micrococcaeae* growth from joint fluid and bone tissue^*a*^

Characteristics	Total (*n* = 13)	True infection (*n* = 4)	Contamination (*n* = 9)	*P* value of difference^*b*^
Age (yr)	67 (45 to 81)	60 (37 to 71)	76 (43 to 83)	0.315
Women	5 (38)	1 (25)	4 (44)	0.506
Charlson score	1 (0 to 1)	1 (0 to 1)	1 (0 to 1)	0.488
Malignancy	0 (0)	0 (0)	0 (0)	
Site of acquisition				
Community	9 (69)	1 (25)	8 (89)	0.021
Nosocomial	0 (0)	0 (0)	0 (0)	
Health care-associated	4 (31)	3 (75)	1 (11)	0.021
Immunosuppressive therapy	4 (31)	2 (50)	2 (22)	0.317
Sepsis or septic shock^*c*^	0 (0)	0 (0)	0 (0)	
Intravascular device^*d*^	3 (23)	1 (25)	2 (22)	0.913
Joint prothesis	5 (38)	1 (25)	4 (44)	0.506
Days of hospitalization as from blood sample taken	1 (0 to 10)	9 (2 to 10)	0 (0 to 7)	0.329
Fever^*c*^	4 (31)	3 (75)	1 (11)	0.021
Leukocytosis^*c*^	2 (15)	2 (50)	0 (0)	0.021
Leukopenia^*c*^	0 (0)	0 (0)	0 (0)	
Mortality	0 (0)	0 (0)	0 (0)	
Polymicrobial growth	4 (31)	0 (0)	4 (44)	0.109
Genus and species				0.853 (genus), 0.375 (species)
Rothia	7 (54)	2 (50)	5 (56)	
R. dentocariosa	3 (23)	0 (0)	3 (33)	
R. mucilaginosa	3 (23)	2 (50)	1 (11)	
Rothia spp. not further specified	1 (7)	0 (0)	1 (11)	
Kocuria	6 (46)	2 (50)	4 (44)	
K. kristinae	1 (7)	0 (0)	1 (11)	
Kocuria spp. not further specified	5 (38)	2 (50)	3 (33)	

a The categorial data are presented as No. (%). Continuous variables are presented as medians (interquartile ranges). For categorial data, the chi-square test is used. For continuous data, the Mann-Whitney *U* test is used.

b Difference between those with true infection and those with contaminated samples.

c Within 48 h from when the blood sample was taken.

d Intravascular devices include implantable cardioverter defibrillators (ICDs), pacemaker, vascular graft, central venous catheter, dialysis catheter.

**TABLE 5:** Clinical and microbiological features of patients with non-Micrococcus *Micrococcaeae* growth from other locations than blood, joint fluid, and bone tissue^*a*^

Characteristics	Total (*n* = 18)	True infection (*n* = 3)	Contamination (*n* = 15)	*P* value of difference^*b*^
Age (yr)	64 (43 to 74)	74	62 (35 to 69)	0.313
Women	9 (50)	2 (67)	7 (47)	0.527
Charlson score	2 (2 to 5)	2	1 (0 to 5)	0.501
Malignancy	5 (28)	0 (0)	5 (33)	0.239
Site of acquisition				
Community	7 (39)	1 (33)	6 (40)	0.829
Nosocomial	5 (28)	0 (0)	5 (33)	1.000
Health care-associated	6 (33)	2 (67)	4 (27)	0.180
Immunosuppressive therapy	3 (17)	0 (0)	3 (20)	0.396
Sepsis or septic shock^*c*^	0 (0)	0 (0)	0 (0)	
Intravascular device^*d*^	8 (44)	3 (100)	5 (33)	0.034
Days of hospitalization from blood sample taken	5 (0 to 18)	16	1 (0 to 10)	0.150
Fever^*c*^	4 (22)	1 (33)	3 (20)	0.612
Leukocytosis^*c*^	4 (22)	0 (0)	4 (27)	0.310
Leukopenia^*c*^	0 (0)	0 (0)	0 (0)	
Mortality	1 (6)	0 (0)	1 (7)	0.645
Polymicrobial growth	0 (0)	0 (0)	0 (0)	
Genus and species				0.105 (genus), 0.206 (species)
Rothia	9 (50)	0 (0)	9 (60)	
R. dentocariosa	3 (17)	0 (0)	3 (20)	
R. mucilaginosa	2 (11)	0 (0)	2 (13)	
Rothia spp. not further specified	4 (22)	0 (0)	4 (27)	
Kocuria	8 (44)	3 (100)	5 (33)	
K. kristinae	2 (11)	0 (0)	2 (13)	
Kocuria spp. not further specified	6 (33)	3 (100)	3 (20)	
Arthrobacter spp.	1 (6)	0 (0)	1 (7)	

a The categorial data are presented as No. (%). The continuous variables are presented as medians (interquartile ranges). For the categorial data, the chi-square test is used. For continuous data, the Mann-Whitney *U* test is used.

b Difference between those with true infection and those with contaminated samples.

c Within 48 h from when the blood sample was taken.

d Intravascular devices include implantable cardioverter defibrillators (ICDs), pacemakers, vascular grafts, central venous catheters, and dialysis catheters.

Four patients had growth of NMM in joint fluid and were diagnosed with septic arthritis. All patients had risk factors for infection: recent knee surgery (*n* =1), knee prothesis (*n* = 1), and immunosuppressive treatment (*n* = 2). The bacteria causing infections in joints were Kocuria sp. and R. mucilaginosa.

A pacemaker infection caused by Kocuria sp. was found in one patient, and peritonitis was found in one patient. One patient was classified as having an infection with isolation of Kocuria sp. in material from an aortic pseudoaneurysm; cultures were taken perioperatively, but the patient did not have any clear signs of infection before surgery.

## DISCUSSION

While previous case reports have identified single cases of infection with Kocuria and Arthrobacter, this study, to our knowledge, represents the first systematic effort to describe what characterizes infection and contamination with these bacteria. Bacteremia with Rothia has been described in a case series (26), but this is, to our knowledge, the largest case series to date and the first to describe invasive infections outside the bloodstream. None of the patients with findings of Pseudoglutamicibacter from cultures turned out to have a true infection, but since this study only covered six cases, it is difficult to draw any conclusions about whether they can cause infections or not. Patients with malignancy seem to be more vulnerable to infections with the studied bacteria. An association between malignancy and Rothia infections has been shown in previous studies, but not in infections with Kocuria and Arthrobacter (26). Even when applying multiple positive blood cultures as the definition of a true infection, the results are similar.

According to this study, findings of Rothia in blood cultures accounted for true infection in 22% of the cases. How often growth of Rothia in a blood culture represents true infection was also analyzed in 2014 by Ramanan et al. (27), describing 67 cases. In that study, a true infection was classified as having multiple positive blood cultures. Rothia grew in multiple blood cultures in 37% of the patients, and leukemia was identified as a risk factor for infection. The proportion of Rothia bacteremia with growth in multiple blood cultures in our material was lower at 13%. Region Skåne, Sweden, had an average of 1.3 million inhabitants between 2012 and 2021, indicating that true bacteremia with the studied bacteria has an incidence of 2.8 cases per 1 million inhabitants per year and that the total incidence of invasive infections is 3.3 cases per 1 million inhabitants per year.

Since many of the studied bacteria are found on the skin, drawing blood from venous catheters, as opposed to directly from the vein, might be more likely to result in contamination (as is the case for other skin contaminants). Information on the method used to draw the blood in individual instances was not available, but the recommendation throughout the study period has been to draw the blood directly from the vein.

TTP was shorter in cultures representing true infection compared with contaminated samples. Former studies have shown that in some bacteria species, a shorter TTP can be an indication of severe infection due to higher bacterial concentration in blood (28, 29). This study shows that shorter TTP in cultures with the studied bacteria is correlated with a higher risk of true infection, but any correlation between TTP and the severity of infection was not possible to confirm.

Regarding the identification of the studied bacteria in locations other than blood, seven cases with true infection were found, including septic arthritis caused by R. mucilaginosa and arthritis, peritonitis, and pacemaker infection caused by Kocuria spp. Except for Kocuria peritonitis, these infections have previously been identified although only published in a few case reports, with each making these findings among the first published (30–34). The adjustments for pediatric blood cultures and for infections outside the bloodstream are somewhat less robust than the ones used for adults with bloodstream infection. However, not taking this into consideration would probably result in a definition with which true infection was overestimated.

A strength of this study compared with other studies is that the cultures were collected at a central laboratory covering all inpatient and outpatient care in the whole region. This makes the result more representative for the population in general compared to a hospital-based design. This is one of the first studies looking at the clinical implications of finding Rothia in cultures in a systematic way and the first systematic study of the clinical implications of finding Kocuria, Arthrobacter, or Pseudoglutamicibacter. This study also has several limitations, mainly due to its retrospective design. Data regarding specific clinical sign were sometimes missing in the medical records, and the amount of blood collected was not documented. The fact that several bacterial species were grouped and analyzed together causes a risk that differences between them are missed. However, the individual species (except for some Rothia species) had few cases despite the long study period, making it difficult to perform statistical analyses on them separately.

In conclusion, Rothia, Kocuria, and Arthrobacter can cause infections and approximately 9 to 22% of all positive blood cultures with these bacteria represent true infections. Growth of NMM in blood cultures should be considered true infections foremost in immunocompromised patients. However, a large proportion of the patients whose charts were reviewed in this study and had a true NMM infection had neither immunosuppressive treatment nor a diagnosed malignancy, showing the importance of combining clinical information and microbiological information such as short TTP in blood culture and lack of other identified likely pathogens when finding uncommon bacteria such as these. These infections seem most often to be subacute, and cases have a favorable prognosis.
